# Cross-recognition of a pit viper (Crotalinae) polyspecific antivenom explored through high-density peptide microarray epitope mapping

**DOI:** 10.1371/journal.pntd.0005768

**Published:** 2017-07-14

**Authors:** Mikael Engmark, Bruno Lomonte, José María Gutiérrez, Andreas H. Laustsen, Federico De Masi, Mikael R. Andersen, Ole Lund

**Affiliations:** 1 Department of Bio and Health Informatics, Technical University of Denmark, Kgs. Lyngby, Denmark; 2 Department of Biotechnology and Biomedicine, Technical University of Denmark, Kgs. Lyngby, Denmark; 3 Instituto Clodomiro Picado, Facultad de Microbiología, Universidad de Costa Rica, San José, Costa Rica; Muséum National d'Histoire Naturelle, FRANCE

## Abstract

Snakebite antivenom is a 120 years old invention based on polyclonal mixtures of antibodies purified from the blood of hyper-immunized animals. Knowledge on antibody recognition sites (epitopes) on snake venom proteins is limited, but may be used to provide molecular level explanations for antivenom cross-reactivity. In turn, this may help guide antivenom development by elucidating immunological biases in existing antivenoms. In this study, we have identified and characterized linear elements of B-cell epitopes from 870 pit viper venom protein sequences by employing a high-throughput methodology based on custom designed high-density peptide microarrays. By combining data on antibody-peptide interactions with multiple sequence alignments of homologous toxin sequences and protein modelling, we have determined linear elements of antibody binding sites for snake venom metalloproteases (SVMPs), phospholipases A_2_s (PLA_2_s), and snake venom serine proteases (SVSPs). The studied antivenom antibodies were found to recognize linear elements in each of the three enzymatic toxin families. In contrast to a similar study of elapid (non-enzymatic) neurotoxins, these enzymatic toxins were generally not recognized at the catalytic active site responsible for toxicity, but instead at other sites, of which some are known for allosteric inhibition or for interaction with the tissue target. Antibody recognition was found to be preserved for several minor variations in the protein sequences, although the antibody-toxin interactions could often be eliminated completely by substitution of a single residue. This finding is likely to have large implications for the cross-reactivity of the antivenom and indicate that multiple different antibodies are likely to be needed for targeting an entire group of toxins in these recognized sites.

## Author summary

Although snakebite antivenom is a 120-year-old invention, saving lives and limbs of thousands of snakebite victims every year, little is known about the mechanisms and molecular interactions of how antivenoms neutralize snake toxins. Antivenoms are produced by immunizing large animals with cocktails of snake venoms resulting in antibodies recognizing toxic as well as non-toxic venom proteins to variable degrees. As a result, high doses of antivenom are needed for treating a snakebite victim, causing more severe adverse reactions due to a high burden of heterologous antivenom proteins. For the first time, we have characterized the antibody recognition sites on hundreds of pit viper toxins using high-throughput peptide microarray technology and an antivenom specific for three pit vipers inflicting a high number of bites in Central America. Most pit viper toxins are enzymes known to have a catalytic site important for toxicity. However, our results suggest that the employed antivenom generally does not target such sites, but instead inhibits toxicity by binding to alternative sites, possibly causing conformational shifts in the toxin structures or interference with toxin-target recognition. The identification of these toxin-specific recognition sites may explain why the antivenom is effective against certain snakebites from pit vipers whose venoms are not part of the immunization mixture.

## Introduction

Snakebite envenoming constitutes a serious public health problem on a global basis [1–3]. It primarily affects impoverished populations living in rural settings of Africa, Asia, and Latin America [4]. It is estimated that about 70,000 snakebite cases occur in Latin America every year, although it is likely that the actual magnitude of the problem is higher owing to the poor records of these accidents in many countries [5].

Parenteral administration of animal-derived antivenoms is the centerpiece of snakebite envenoming therapy. In Latin America, several laboratories are manufacturing antivenoms against the most relevant venomous snake species [6,7]. The vast majority (> 95%) of envenomings in Latin America are caused by species classified in the family Viperidae, subfamily Crotalinae, commonly referred to as pit vipers [5]. Most antivenoms against pit viper envenomings are polyspecific, meaning that venoms from more than one species are used in the immunization process. The resulting antivenom is therefore effective against bites from a range of snake species. This is crucial owing to the difficulty of species identification upon a snakebite. In Central America and Mexico, polyspecific antivenoms are produced by immunizing horses with mixtures of venoms of *Bothrops*, *Crotalus*, and *Lachesis*. In general, polyspecific antivenoms manufactured in various Latin American countries have shown ability to cross-neutralize several heterologous venoms, i.e. venoms not used in the immunization schedule. This phenomenon is referred to as para-specificity, and is especially prominent for species that belong to the *Bothrops* genus (lance-headed vipers) [8–12]. However, para-specific antigenic recognition and neutralization of venoms is not always observed at the intra-generic level, and cannot be assumed *a priori* only on the basis of taxonomy [13,14]. For venoms of the American *Micrurus* elapids (coral snakes), a marked antigenic divergence has been documented, where antivenoms raised against particular species failed to cross-neutralize congeneric venoms [15–19]. Similarly, cases of antigenic divergence leading to lack of cross-recognition of toxins among venoms of viperid species have been described, although these are mainly explained by the existence of certain toxins that are not widespread across all taxa. For example, the polyspecific Crotalinae antivenom prepared in Costa Rica using venoms of *Bothrops asper*, *Crotalus simus*, and *Lachesis stenophrys* [20] as immunogens, cross-neutralizes the venoms of the local *Bothriechis* (palm vipers) species (*B*. *lateralis*, *B*. *schlegelii*, *B*. *supraciliaris*), except for *B*. *nigroviridis*. The venom from the latter species contains a high proportion of a lethal 'crotoxin-like' phospholipase A_2_ (PLA_2_) named nigroviriditoxin, which is not cross-recognized by antivenom antibodies targeting heterologous PLA_2_s [21].

Para-specificity of an antivenom has traditionally been assessed by *in vivo* studies in mice and supported by a variety of immunological techniques. In the more recent years, a standardized method, referred to as “antivenomics” [22], combining affinity chromatography with proteomic identification of antigens, has gained widespread acceptance. Overall, the information provided by such immunological analyses reveals if cross-recognition occurs between venom components on a protein family level, indicating the existence of antibody recognition sites (epitopes) shared between heterologous toxins.

To gain molecular level insight into para-specificity, antivenom cross-recognition of individual toxins can be assessed using synthetic peptides representing linear elements of B-cell epitopes on the toxins [23,24]. Despite the inability to evaluate discontinuous epitopes, a growing number of studies have proven the usefulness of linear epitope analyses in antivenom research. Linear elements of epitopes have been found and the ability of synthetic mimicking peptides to induce a neutralizing antibody response have been demonstrated, opening new possibilities to improve the efficacy of snakebite antivenoms in the near future [25–31]. Due to technological limitations of traditional peptide synthesis and cellulose-bound peptide arrays (spot-synthesis) and the high number of overlapping peptides needed to perform such meticulous experiments, reported studies have each focused on no more than five toxins. However, by harnessing high-density peptide microarray technology, we recently enabled high-throughput molecular level study of para-specificity of toxins and antivenoms by characterizing epitopes in 82 related toxins from African *Dendroaspis* (mamba) and *Naja* (cobra) species [32].

In this study, we scaled up the high-density peptide microarray method to identify linear elements of epitopes in 702 pit viper toxins and 168 partial toxin sequences obtained from the UniProtKB database [33], using the Costa Rican polyspecific Crotalinae antivenom as probe [34]. Even though sequence data is only available for 69 of the 151 described pit viper species, the large scope of the study allows in-depth characterization of linear elements of epitopes. With its high number of investigated toxins and broad species coverage, this study is thus the largest of its kind performed to date.

## Methods

### Antivenom and negative control preparation

The polyspecific Crotalinae antivenom (hereafter called ‘antivenom’) analyzed here (batch 5500914POLQ, expiry date: September 2017) was produced at Instituto Clodomiro Picado, University of Costa Rica, by immunization of horses with a mixture of *Bothrops asper*, *Crotalus simus*, and *Lachesis stenophrys* venoms as described elsewhere [20]. An immunoglobulin preparation obtained from the plasma of non-immunized horses, processed in the same manner as the antivenom, was used as a negative control. The antivenom and the plasma of non-immunized horses were not prepared specifically for this study.

### Design of high-density peptide microarrays

An *in silico* library of peptides was generated to span the entire length of the 702 pit viper toxins and 168 partial toxin sequences that were available in the UniprotKB database at the time of the microarray design (February 2015). The library consisted of 174,797 15-mer peptides derived from the primary sequences of each toxin by displacement of the running window by one amino acid residue allowing overlap of 14 residues for neighboring peptides. The four toxin sequences containing unspecified residues had such residues replaced with glycines in their sequences prior to generation of peptides. The array of peptides was curated for redundant (non-unique) peptide sequences, leaving 82,423 unique 15-mers, which were included in five replicates. The individual peptides in the library were assigned random positions on the microarray to minimize local intensity biases.

### Peptide microarray hybridization

The peptide microarray was produced by Schafer-N (Copenhagen, Denmark) using mask-less photolithographic synthesis adapted to solid-phase peptide synthesis with the C-terminal residue linked to the surface of the array, as previously described [35]. The microarray was first incubated for 1.5 hours with 50 μg/mL naïve horse IgG in 0.05 M Tris/acetate (Trizma base, Sigma-Aldrich), pH 8.0, 0.1% v/v Tween 20, 1 g/l Bovine serum albumin (dilution and washing buffer). After washing, the array was incubated with 1 μg/ml of goat anti-horse IgG (H+L) conjugated with AlexaFluor 647 (Jackson ImmunoResearch, 108-605-003) at room temperature for 1.5 hours. Washing procedure was repeated to remove unbound conjugates and an image was recorded using an InnoScan900 microarray scanner (Innopsys) with an excitation wavelength of 635 nm. The microarray was then washed and incubated with 50 μg/mL antivenom for 1.5 hours, followed by further washing and a second incubation with the same antibody conjugate as above, washed and recorded. Fluorescence intensity for each peptide field was calculated from the resulting image files using proprietary software by Schafer-N.

### Identification of antibody binding peptides

The 15-mer peptide KKKRKKKRKKKRKKK was synthesized in 852 fields and used to define the corners of the microarray grid, as this peptide is highly prone to unspecific antibody binding. The effect of two successive binding events for peptides prone to unspecific binding was determined using the signals from corner peptide fields as the difference in the median signal in each recording. This re-incubation effect results from interaction of antivenom antibodies with free binding sites on bi-valent secondary anti-horse antibodies already attached to naïve IgG and additional binding of antivenom antibodies to free peptides on the microarray surface. The re-incubation effect was found to provide 4.3 times higher signals in the second incubation than in the first. To remove such artefacts, the antivenom signal was subtracted by the background signal multiplied by the re-incubation effect.

Aiming at reducing the impact of outliers on the downstream data analysis, the median signal of five replicates of each peptide was determined after both binding of naïve IgGs and antivenom IgGs. The signal medians were mapped to each toxin by the position of the N-terminal residue in the original protein sequences. As linear elements of epitopes are usually between 4 and 12 amino acid residues in length [35], true epitopes result in high signals across overlapping peptides. Therefore, the running median of signals was calculated by taking the signals of the nearest preceding and subsequent 15-mer peptide into account.

The likelihood of each signal to be a result of specific antibody recognition was determined using the one-sided Z-test, assuming the corrected running median of random peptides follows a normal distribution (with mean of 0). The standard error of five replicates was found to increase with signal strength, which is why a conservative estimation approach for the standard error of the running median was followed, using the signal intensities of corner peptides as they showed a high level of unspecific antibody binding. By random sampling with replacement (bootstrapping) from the two empirical distributions of signal intensities of corner peptides (background and antivenom), the standard deviation of the background-corrected running median score was determined. The sampling process was repeated 50,000 times, resulting in a standard deviation of 12.31. The results for each unique peptide-running median pair (n = 84,023) were corrected for multiple comparisons using Benjamini-Hochberg procedure for controlling the false discovery rate and significance threshold was set to α = 0.05, resulting in a significant corrected running median score to be above 37.94. This number is referred to as the significance threshold.

### Analysis of linear epitopes across protein subfamilies

Protein family affiliation for each toxin was obtained from UniprotKB and a multiple sequence alignment of each protein subfamily was constructed using Clustal Omega [36]. The corrected running median scores were mapped to the alignment using the position of the N-terminal residue of each 15-mer. In case of alignment gaps, no data was attached to the given positions for corresponding toxins. The corrected running median scores assigned to positions in multiple sequence alignments were visualized as signal profiles [32], and any peaks above the significance threshold and present across more than ten toxins were investigated further. The toxin with the highest peak was used to identify overlapping 15-mer peptides of relevance to an epitope. The core motif of the linear epitope was extracted and the sum of scores of all 15-mers containing the motif was determined. This procedure was performed for similar 15-mer peptides across all toxins in each multiple sequence alignment. A peptide was considered similar to a recognized peptide if no more than three residues were different between the pair. The resulting core sequences, including sum of scores, were analyzed using the SigniSite 2.1 algorithm to identify residues associated with high or low sum of scores [37]. Like above, the significance threshold was set to α = 0.05. The results were illustrated as sequence logos using the R “ggseqlogo” package [38].

### Mapping of linear epitope elements to protein models

The core residues found across each series of recognized overlapping peptides were assigned a value corresponding to the sum of running median scores obtained for the given peptides. The values were mapped using the R “Rpdb” package to a crystal structure pdb-file (when available), or alternatively to a homology model constructed using CPHmodels [39]. Metalloprotease-domains: P83512 from *B*. *asper* mapped to RCSB entry: 2w15 [40]; J3SDW8 from *C*. *adamanteus* homology model based on RCSB entry 2ERQ [41]. Serine proteases: Q072L7 from *Lachesis stenophrys* and J3S832 from *Crotalus adamanteus* homology models based on RCSB entry 2AIP [42].

## Results and discussion

### Linear elements of epitopes were detected in half of the investigated toxins in a protein subfamily-dependent manner

**Fig 1:**
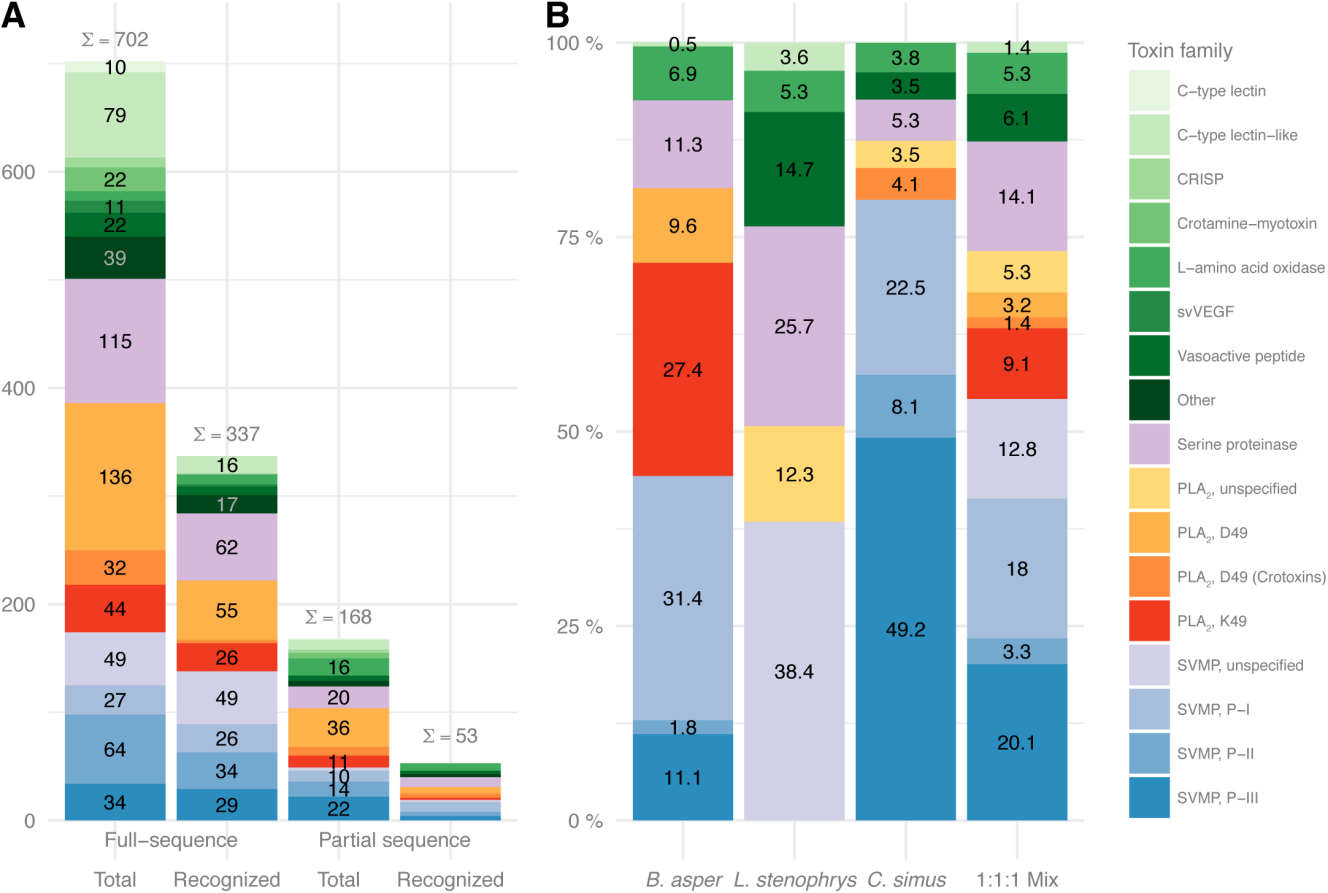
Protein family relationships for toxins in this study and in venoms used in antivenom production. (A) Overview of the investigated toxin sequences specifying the protein family relationship and if the sequences are classified as partial or full-sequences covering entire toxins. The numbers within the bars represent counts of ten and above. Sums (∑) of all counts are shown as on top of each stacked bar. (B) Percentage distributions of protein families in venoms used in production of the antivenom. Numbers within the bars represent dry weight percentages for each protein family relative to the total protein content as determined in venomics studies: *B. asper* [43], *L. stenophrys* [44], and *C. simus* [45]. Comparing the immunization mixture composed by equal amounts of each of the three snake venoms (bar no 4 in B) with A shows that all major protein family components in the immunization mixture are represented in the peptide microarray.

Aiming at gaining molecular insights into antivenom para-specificity, linear elements of epitopes were identified using a custom designed high-density peptide microarray. The setup included overlapping 15-mer peptides from the 702 full-sequence pit viper toxins available in UniprotKB at the time of the experiment. The microarray was designed similarly to a previous study of mamba and cobra toxins [32]. The median signal intensity of each peptide was determined and mapped to the toxin sequence. Potential linear epitope elements were identified when the signal of two successive 15-mers was found to pass the significance threshold (see method section for details). Following this approach, at least one linear epitope element was identified in 337 out of 702 full-sequence toxins. Furthermore, linear epitope elements were identified in 53 out of 168 toxin sequences classified as incomplete, meaning that linear elements of epitopes might exist in the un-sequenced (and therefore not investigated here) parts of the toxins. Segmented into protein families, the results are summarized in Fig 1A.

Proteomics-based “venomic” studies of pit viper venoms have revealed that their most abundant toxins belong to a limited number of protein families. In general, the main toxic effects exerted by Latin American pit viper venoms are caused by zinc-dependent snake venom metalloproteases (SVMPs), phospholipases A_2_ (PLA_2_s), and snake venom serine proteases (SVSPs), although other components may contribute to the overall toxicity [46–49]. These less predominant toxin families include cysteine-rich secretory proteins (CRISPs), C-type lectin and lectin-like proteins, L-amino acid oxidases, vasoactive peptides, and crotamine in the case of some *Crotalus* (rattlesnake) species [50,51]. Members of each of the mentioned protein families are represented in this study, however, the detailed epitope characterization in the following will focus on the 501 full-sequence toxins and 124 partial toxin sequences from the three major protein families.

The three snake venoms used in the immunization mixture for production of the antivenom are well-described on the protein family level [43–45] as illustrated in Fig 1B. However, only 11 complete toxin sequences and 4 partial sequences (8 being full sequences and 4 partial sequences from *B*. *asper*, 3 full sequences from *L*. *stenophrys*, and none from *C*. *simus*) were available in the UniprotKB database, when this study was designed (the toxin entry C0HK50 from *C*. *simus* was added afterwards and is not part of the study). This limitation on antigen sequences is compensated by the inclusion of a vast number of homologous sequences in each of the medically relevant protein families (see Fig 1A).

**Fig 2:**
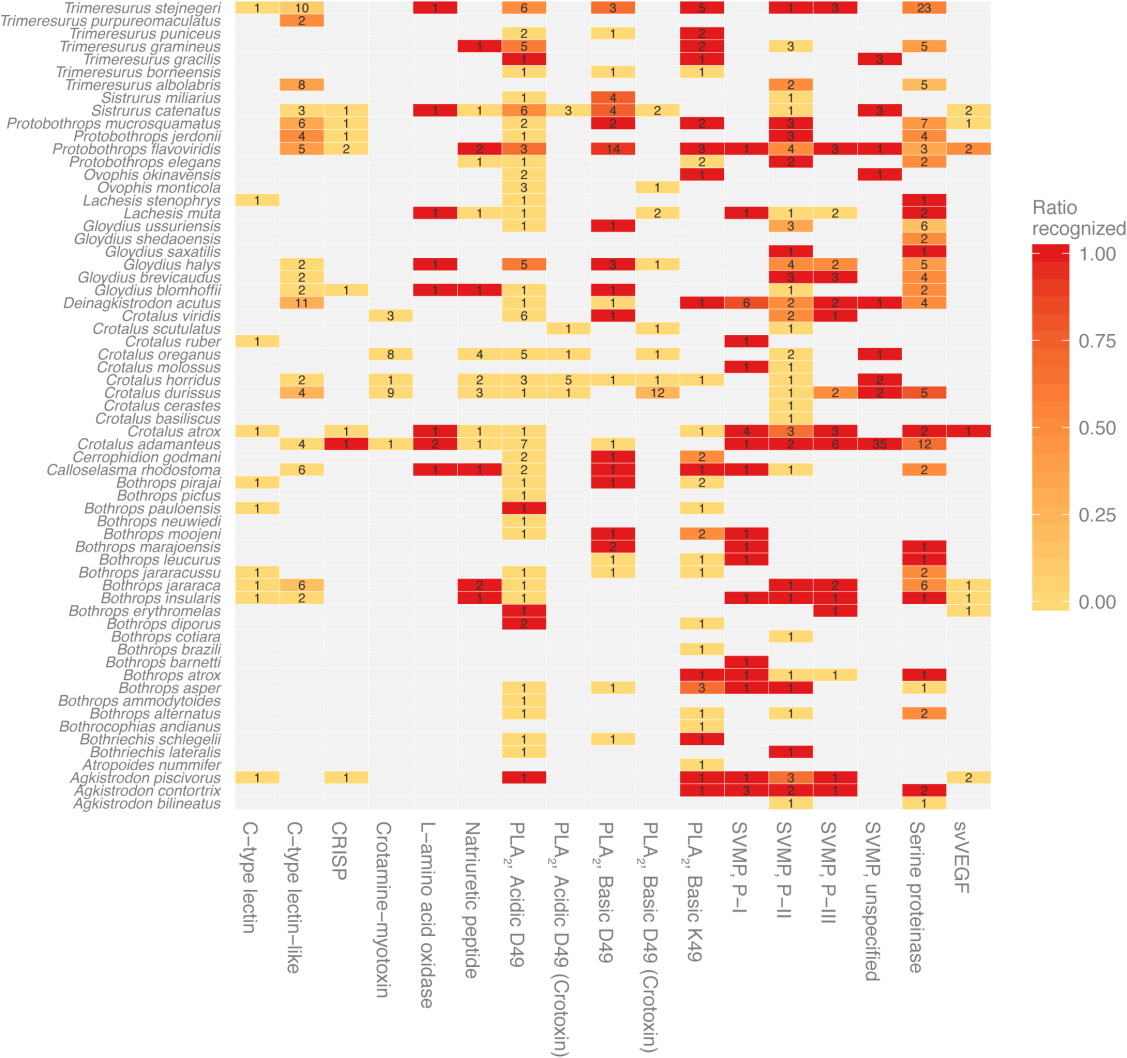
Antivenom recognition of full-sequence toxins grouped by species and protein family. Toxins of different protein families and from different species were not recognized equally well by the antivenom. The number in each tile refers to the number of investigated toxins. Tile color represents the ratio of toxins recognized by the antivenom as determined by significant binding of one or more toxin-derived peptides: Dark red corresponds to recognition of all toxins in the group; pale yellow corresponds to no recognized toxins.

Imposing information of the snake genera to the results for the full-sequence toxins in Fig 1A results in a more detailed overview of para-specificity for the antivenom (Fig 2). In this overview, each group of toxins from the same species and protein family (or subgroup when applicable) is colored to reflect the proportion of toxins for which one or more linear elements of an epitope were identified.

In some toxin families, no linear elements of epitopes were identified (e.g. the 22 members of the crotamine family), while all members of e.g. the nine L−amino acid oxidase family had linear epitope elements that were recognized by the antivenom. As an epitope is a surface area in three-dimensional space, an epitope might not contain sufficiently long linear parts to be detected in this setup. This also means that no recognition of peptides from e.g. the crotamine family does not prove that crotamine-specific antibodies are absent or that the antivenom will display poor clinical efficacy against venoms containing these toxins. However, poor retention of crotamines has previously been reported in antivenomic studies with the same antivenom and is congruent with the absence of crotamine in the venom of *C*. *simus* from Costa Rica used in the horse immunization procedure for antivenom manufacture (Fig 1B) [45]. In general, the lack of peptide interactions with the antivenom antibodies might be due to absence of common linear elements between different toxin epitopes or due to low immunogenicity of whole groups of toxins.

### The antivenom recognizes several sequence segments centered around a small α-helix in the metalloprotease domain distant from the enzymatic site

**Fig 3:**
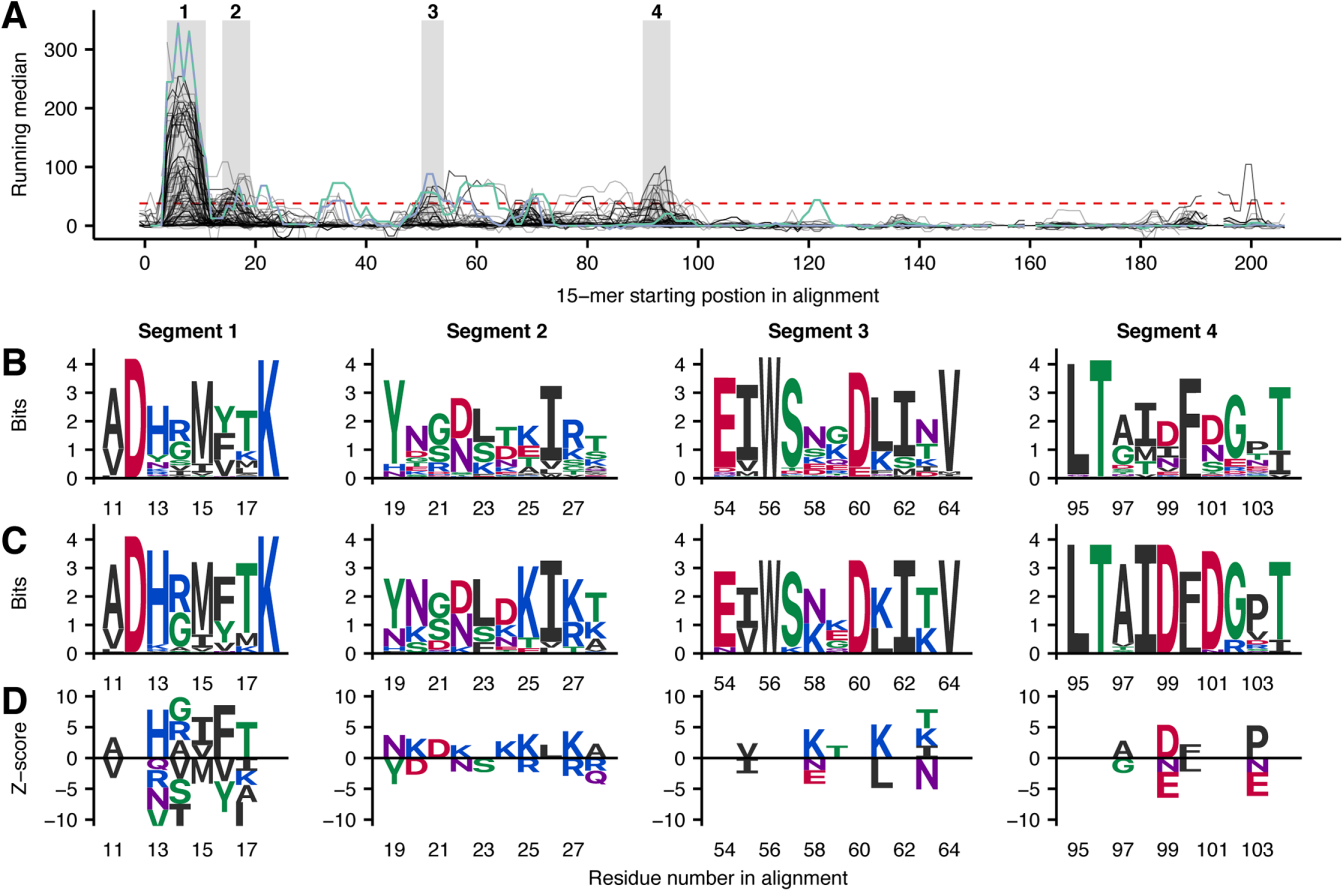
Representations of linear epitope elements in M-domains of SVMPs. (A) Mapping of signals from antibody recognition of peptides to the primary sequence of 222 M-domains of SVMPs (49 labelled as partial sequences). The signals are mapped to a multiple sequence alignment, corrected for unspecific binding, and represented by a running median score. The red, dashed horizontal line indicates the threshold for a score to be significantly higher than background noise. Segments with more than ten toxins crossing the significance threshold are marked in grey boxes. Positions containing gaps for all but one toxin were removed from the signal plot to make it simpler to read, and as there was no significant signals observed for the given peptides. The green signal profile is P83512 and the blue signal profile is Q072L5 (both from *B. asper*). (B) Sequence logos (Shannon type) representing the overlap between peptides from all toxins in each segment of the multiple sequence alignment. The height of each letter is equal to the information content (log likelihood ratio) of each amino acid in a position in bits. This can be understood as degree of conservation of each residue. (C) Sequence logos (Shannon type) solely representing toxins recognized in a segment. (D) Z-score sequence logos showing SigniSite [37] output. Amino acid residues on the positive y-axis are associated with higher signals, while residues on the negative y-axis are associated with weak or no binding. Only residues with a significance level of α = 0.05 are shown.

The protein family of SVMPs is known to be among the key toxins responsible for the toxicity of pit viper venoms and these enzymes are known to induce degradation of collagen in the vascular basement membrane resulting in local hemorrhage, as well as in other local and systemic pathological effects [48,52]. The binding data for each of the 223 (174 full sequence) SVMPs was aligned to obtain a holistic understanding of recognition by the antivenom antibodies. Due to variations in domain composition between the individual subfamilies of SVMPs, each domain was investigated separately. The hallmark metalloprotease (M) domain is omnipresent in immature form of all SVMP family members. However, disintegrins and DC fragments derived from some SVMP P-II and P-III subgroups members do not contain the M-domain in mature form, since it is cleaved post-translationally [53]. Consequently, only 167 sequences (full and partial combined) of M-domains were available for this study. Alignment of the resulting background-corrected running median scores for each 15-mer peptide results in the signal profile in Fig 3A. Here, four segments (highlighted in gray boxes) in the first half of the signal profile were found to contain a peak shared between ten or more sequences.

Two of the M-domains originate from *B*. *asper* venom employed in the immunization mixture: A P-I (P83512) and a P-II (Q072L5) SVMP. The signal plots of the *B*. *asper* toxins (blue and green line in Fig 3A) show high signals in all the highlighted segments except for segment 4. Furthermore, *B*. *asper* toxins are recognized at four additional sites, which are not commonly shared by other M-domains and therefore not discussed here. As these two toxins were present in the immunization mixture, the finding that they are among the best recognized M-domains is expected and can be regarded as support for the methodology.

Multiple sequence alignments of the core residues of the overlapping peptides in each of the four selected segments in Fig 3A are represented as sequence logos in Fig 3B. These sequence logos show the general level of residue conservation across the investigated toxins. Only representing the sequences of toxins recognized by the antivenom, the sequence logos in Fig 3C contain the residues that are most likely to play a role as part of epitopes. For segments 1, 3, and 4, a high level of conservation is observed for several positions in the sequence logos, indicating that antivenom recognition and thereby cross-recognition is easily lost when the residues are substituted. This stands in contrast to segment 2, where the antivenom antibodies recognize multiple different motifs.

Using non-recognized toxin sequences similar to the ones recognized by the antivenom, the effect of naturally occurring amino acid substitutions in the investigated positions can be examined. As a measure representing the level of antivenom binding to each toxin sequence, the sums of the running median signals for the series of overlapping peptides were determined. Hereafter, the SigniSite 2.1 algorithm [37] was applied to perform residue level genotype-phenotype correlations and thereby identify amino acid residues significantly associated with lack of antibody binding. The resulting Z-scores obtained for each residue in each position reflect the strength of residue association with either high or low antivenom binding. The Z-scores represented as special sequence logo are found in Fig 3D. In such sequence logo, conserved residues–which might also be essential to antibody recognition–will not show, as such residues are found both in the top as well as in the bottom of the list of peptides ordered according to the determined score by the SigniSite algorithm. Consequently, Fig 3D must be viewed together with Fig 3C to obtain comprehensive understanding of the binding preferences of the antivenom antibodies.

**Fig 4:**
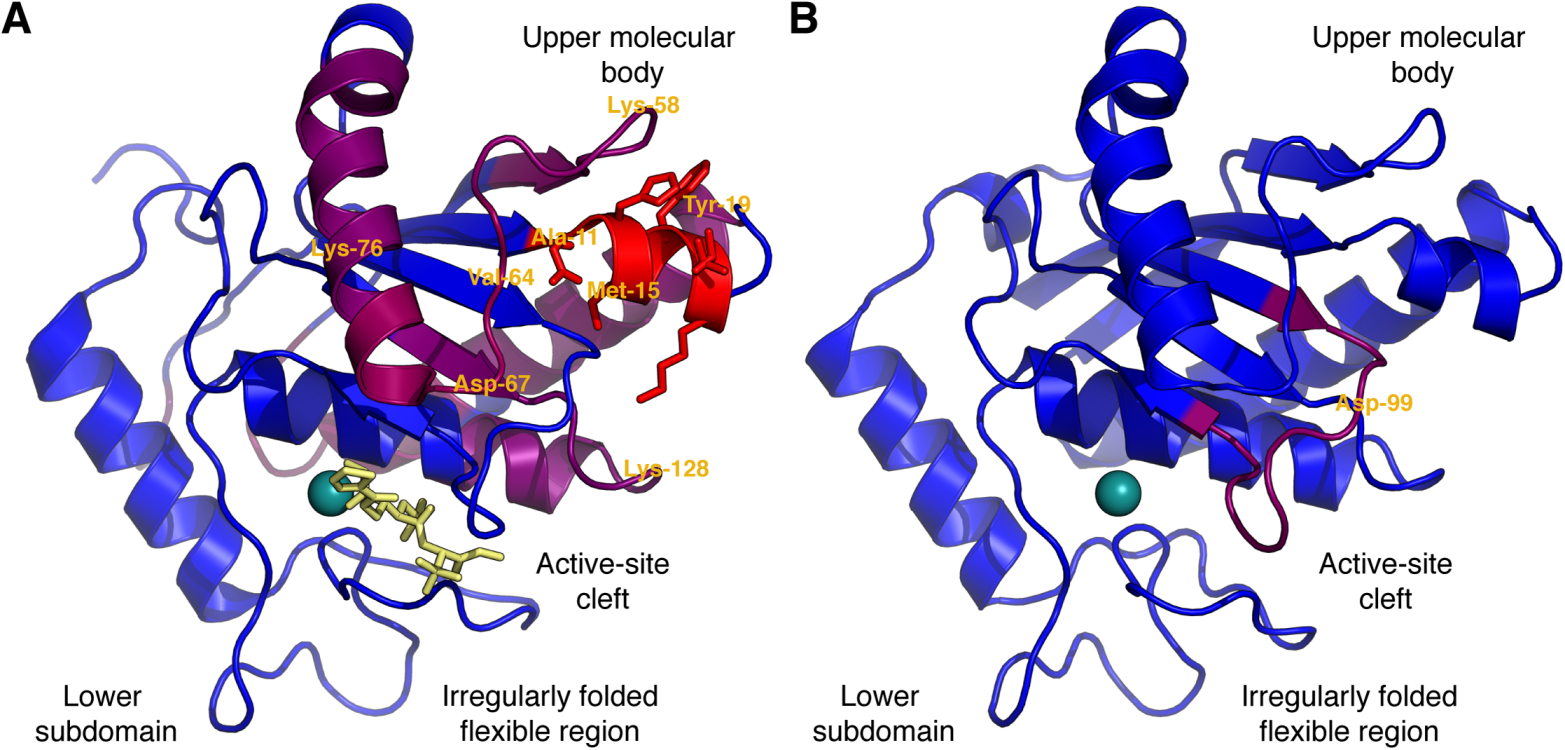
Structural presentation of linear epitope elements found in two representative M-domains. Core residues of each series of overlapping peptides recognized by the antivenom are colored according to the sum of running median scores for the peptide series in a scale from purple to red (largest sum). The rest of the protein backbone is colored blue. Selected residues are labeled for reference to Fig 3. Antivenom antibodies recognize residues in the upper molecular body, while substrate binding is determined by the flexible region in the lower subdomain, and the active site is present between the two subdomains [53]. (A) M-domain from the SVMP P-I, P83512, from *B. asper*. (B) M-domain from the SVMP P-III, J3SDW8, from *C. adamanteus*.

Mapping of the identified linear epitope elements discovered across SVMP M-domains to three-dimensional structures can reveal details about possible mechanisms of neutralization. Several crystal structures of the previously mentioned P-I SVMP from *B*. *asper*, P83512, are available [40]. The core residues found across each series of recognized overlapping peptides were assigned a value corresponding to the sum of running median scores obtained for the given peptides and mapped to a crystal structure of P83512 in Fig 4A. The structural mapping reveals that the recognized residues are proximally located to each other in space and arranged around a small α-helix (commonly referred to as α-1) containing the residues corresponding to the highly-recognized segment 1 in Fig 3. The position in the so-called “upper main molecular body” of the M-domain is far away from both the active site cleft (see also annotations in Fig 4A) and the irregularly folded flexible region in the C-terminal “lower sub-domain”, known to be important for substrate recognition [54,55], and which has been suggested to determine the hemorrhagic potential of some PI SVMPS [56]. Similar to the other three sequence segments, the spatial position of segment 4 in Fig 3 (not recognized in P83512) was also mapped to the upper main molecular body (Fig 4B) and not to parts of the M-domain directly involved in catalysis.

Identification of linear elements of one or more overlapping epitopes distant from the catalytic site might possibly be of limited therapeutic relevance. It is in principle possible that neutralizing epitopes may exist that have too short linear elements to be detected in this analysis. However, three of the linear epitope elements identified here have previously been found to be neutralizing epitopes when studied in rabbits immunized with either isolated P-I SVMPs or mixtures of epitope-mimicking peptides: A study investigating a P-I SVMP from *Lachesis muta* (P22796) also identified segment 1 and 2 as important for antivenom recognition [29]. Immunization with three 12-mer peptides, of which none were overlapping with the catalytic site and one contained segment 1, was sufficient to produce neutralizing antibodies. Furthermore, the second 12-mer peptide corresponded to a non-significant peak centered around 15-mer number 69 in the signal profile for P22796. A similar study on a P-I SVMP from *Bothrops atrox* (P85420) determined segments 2 and 3 to be sufficient in raising a protecting antibody response against the toxin when rabbits were immunized with the corresponding synthetic peptides [31]. In both studies only immunization with combinations of peptides were reported.

Binding of antibodies to the four segments distant from the active site may neutralize enzymatic activity and henceforth toxicity by the following hypothetical mechanisms: 1) Steric hindrance, i.e. binding of a large 150 kDa antibody sterically hinders the much smaller 22 kDa M-domain from interacting with collagen (or other relevant) substrate. Such effect can be large when binding at exosites important for interaction with the relevant tissue target [57,58]; 2) Allosteric effect, i.e. antibody binding at distant sites alters the conformation of the toxin, thus inactivating the enzyme by distortion of the active site. This has been documented for an antibody targeting the upper molecular body of a human membrane metalloproteinase [59]; or 3) Since antibodies are bivalent, the binding may induce cross-linking of several toxin molecules, thus precluding them from reaching or interacting with their targets. Alternatively, cross-linking can lead to formation of larger protein complexes that are more easily cleared by the victim’s immune system. Additionally, antibody binding will also have a profound effect on toxin pharmacokinetics, which may lead to a reduction in toxicity.

Looking at the individual linear elements of epitopes, segment 1 of the aligned signal profiles in Fig 3A (corresponding to the red helix in Fig 4A) shows a very high level of antivenom recognition. 102 out of 168 toxin sequences had one or more peptides above the significance threshold in this segment. Of the eight residues making up the linear epitope element, only two are found not to be exposed at the surface of the toxins (Fig 4A), namely the residues in position 11 and 15. These residues might still be important for forming the helical structure, explaining why only a very limited selection of hydrophobic residues is found in the positions across all toxins (Fig 3B). From the sequence logo in Fig 3C, representing only the recognized toxins in the alignment, very few substitutions were tolerated by the corresponding antibodies. However, many of the residues in the helix were also rather conserved (Fig 3B). This finding can potentially explain the para-specificity of the antivenom and why the antivenom has previously been found to bind all or most P-I and P-III SVMPs from several investigated American pit viper venoms [34]. However, even within the *Bothrops* genus, which generally contains an “ADHR(M/I)FTK” motif in segment 1, non-recognized versions of M-domains violating the pattern exist. This means that having origin from a *Bothrops* snake is not sufficient for a toxin to contain an antibody binding version of segment 1 (although it is likely). As a result, para-specificity of the antivenom to this specific segment of the M-domain is a property which cannot be predicted simply based on phylogenetic relationship.

In the remaining three segments in Fig 3B between 13 and 33 toxins are recognized. Of particular interest, none of the two *B*. *asper* M-domains (Fig 3A) or in fact any M-domains originating from *Bothrops* or *Lachesis* species are recognized in segment 4. Here, the top scoring toxins originate from *Gloydius brevicaudus* and *Crotalus adamanteus* (example in Fig 4B), pointing at a likely origin of this linear epitope element to result from antibodies targeting *C*. *simus* SVMPs. The difference in the antibody recognition profiles between the individual toxins highlights that the expanded “epitope recognition space” of the antivenom obtained from immunizing with a broad range of related toxins (multiple venoms) reduces the likelihood that any given toxin of the same family “goes unnoticed” by the antivenom. However, it is interesting that all identified linear epitope segments are located around the small helix constituting segment 1, far away from the enzymatic site involved in toxicity and tissue damage. This contrasts with elapid neurotoxins, where binding of antivenom antibodies was detected at the functional site [32]. Even though the link to neutralization must be explained by other factors as discussed above, the current findings shine light upon possible mechanisms for para-specificity of the antivenom.

### Peptides from the disintegrin and cysteine-rich domains of SVMPs are poorly recognized by the antivenom

**Fig 5:**
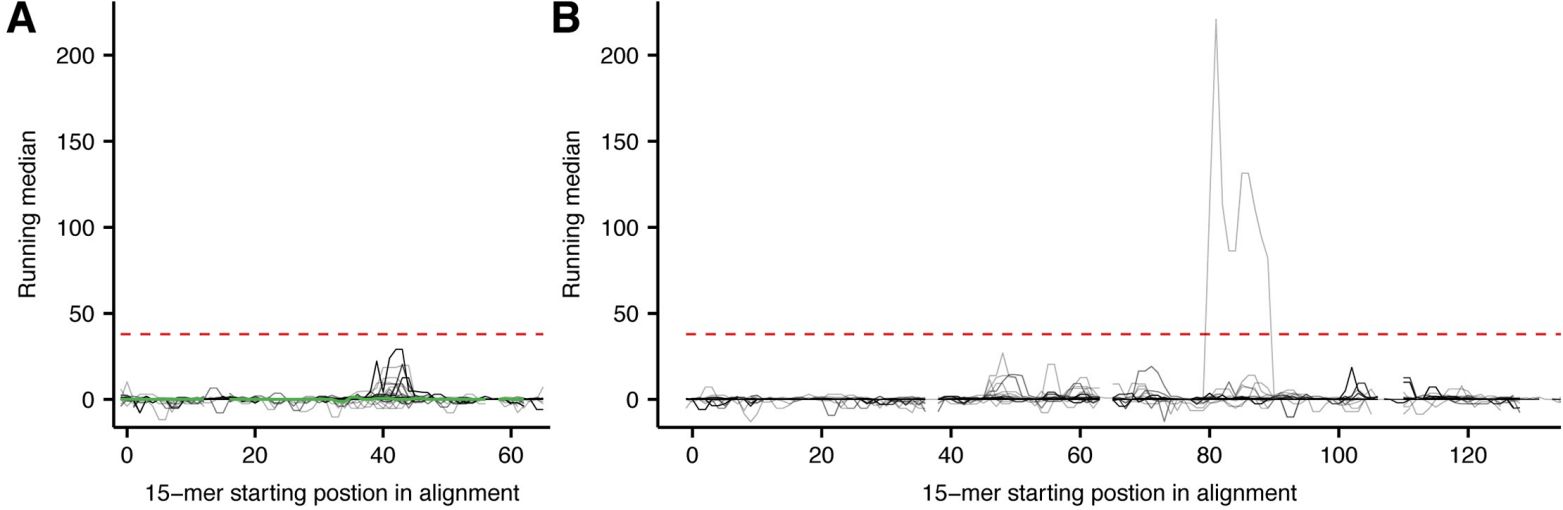
Aligned signal profiles for disintegrin and cysteine-rich domains. Mapping of signals from antibody recognition of peptides to the primary sequence of (A) 179 D-domains (33 labelled as partial sequences) and (B) 117 C-domains (22 labelled as partial sequences). The signals are mapped to multiple sequence alignments, corrected for unspecific binding, and represented by a running median score. The red, dashed horizontal line indicates the threshold for a score to be significantly higher than background noise. The D-domain from *B. asper* toxin Q072L5 is highlighted in green in A. No D-domain derived peptides are recognized by the antivenom, while only peptides from one C-domain sequence are binding the antivenom antibodies.

To understand para-specificity of P-II and P-III SVMPs, it is not sufficient to investigate the M-domain alone. Nonetheless, the binding data for the disintegrin(-like) (D) domain present in both P-II and P-III and the cysteine-rich (C) domain in P-III SVMPs shows only one linear epitope element above the significance threshold (Fig 5). Based on previous reports [34] and the observation that no significant signals were seen for the D-domain sequences, we conclude that disintegrins are likely to be of low immunogenicity. Since the vast majority of PII SVMPs are cleaved post-translationally to release disintegrins [60], and the M-domain in these proteins is degraded, horses receive poorly immunogenic, low molecular weight disintegrins instead of whole PII SVMPs when immunized with the venom.

One C-domain, originating from the mRNA transcript sequence B0VXU4 from *Sistrurus catenatus edwardsii*, is strongly recognized with running median signals above 200 in the area outlined by 15-mer number 80–89 (Fig 5B). The core sequence FCFPNK is unique to this entry. However, from a BLAST search against all toxins of the study, the similar permutated sequence FFCPNK is found as part of a recognized sequence in several SVSPs and the shorter sequence FPNK is found in the likewise well-recognized C-terminal of the acidic D49 PLA_2_ (P84651) from *Lachesis stenophrys* (venom included in immunization mixture). In conclusion, the recognition of B0VXU4 is likely to be a stochastic event resulting from immunization with a similar sequence in another context.

Taken together, the results for the M-, D- and C-domains indicate that SVMP P-III group members are recognized almost exclusively at the M-domain part. However, as epitopes without sufficiently long linear elements to be identified in this analysis may potentially exist, and as some SVMP P-IIIs are post-translationally modified, additional recognition of the D- or C-domain cannot be excluded. The most extreme post-translational modification is observed for the subgroup P-IIId, where a snake venom C-type lectin-like domain is added. Nonetheless, limited antibody binding is also observed for the C-type lectin-like toxins investigated in this study (See overview in Fig 2).

### Phospholipase A_2_s are recognized at three sequence segments

PLA_2_s are found in all pit viper venoms as well as in many other viper and elapid snake venoms. Neutralization of this toxin family is generally very important for an antivenom to be effective in the clinical setting. For an antivenom to have broad coverage across several pit viper venoms, PLA_2_ recognition by the antivenom antibodies must be maintained when variations among the individual toxin sequences exist. Aiming at understanding antivenom recognition of PLA_2_s, 267 PLA_2_ sequences (of which 212 are full sequences) were included in the experimental setup, and a residue-level investigation of antibody binding was performed, resembling the analysis of SVMPs in the previous section.

**Fig 6:**
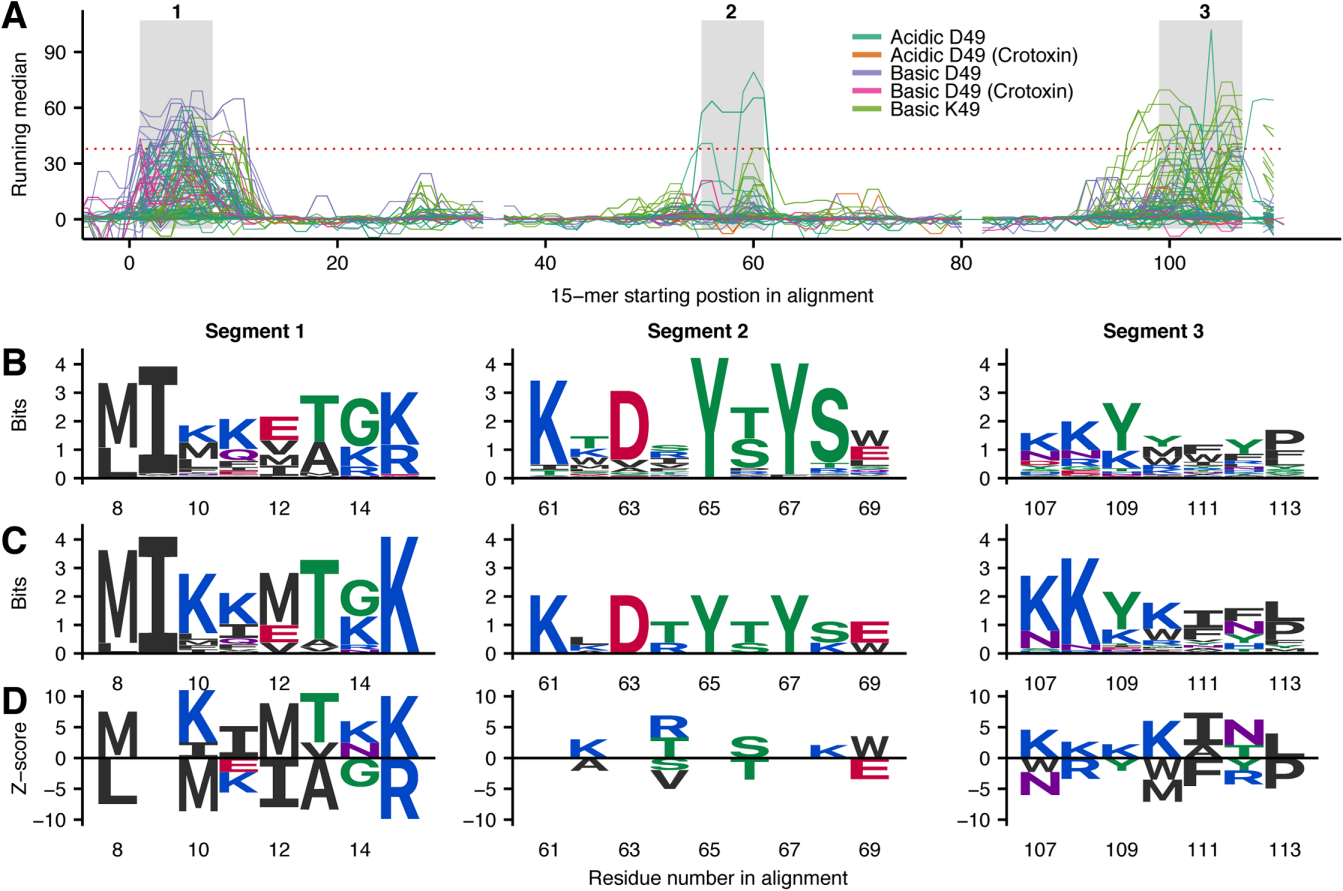
Representations of linear epitope elements in PLA_2_s. See Fig 3 legend for detailed explanation. (A) Three separate segments subject to antivenom recognition are identified in the primary sequences of 270 PLA_2_s (58 labelled as partial sequences). The signal profiles are colored according to the group classification of each toxin showing that different groups dominate the peaks in each segment. (B) Sequence logos (Shannon type) representing the overlap between peptides from all toxins in each segment of the multiple sequence alignment. (C) Sequence logos (Shannon type) solely representing toxins recognized in a segment. (D) Z-score sequence logos showing SigniSite [37] output.

Prior to the analysis, the PLA_2_s were divided into five groups based on the descriptions of the well-characterized full-sequence toxins and clustering of all PLA_2_ sequences, where unannotated sequences were grouped together with similar sequences. The groups include the acidic and basic catalytically active PLA_2_s, which both have a conserved calcium-coordinating aspartic acid in position 49 (D49 groups). The third group consists of basic PLA_2_-like toxins, which have the aspartic acid crucial for enzymatic activity replaced, typically with a lysine in position 49. Therefore, this group is named the K49 group of myotoxins [61]. The neurotoxic “crotoxin(-like)” D49 PLA_2_s, which contain acidic and basic subunits, were grouped independently from the other acidic and basic D49 PLA_2_s, resulting in a total of five groups. The antivenom was found not to recognize linear epitope elements of the individual groups of PLA_2_s equally well (see overview in Fig 2 and Fig 6A). The basic D49 and K49 groups were recognized reasonably well, while the acidic D49 and the neurotoxic crotoxin(-like) PLA_2_s were recognized to a much lower degree. This general finding can be explained by the composition of the immunization mixture (Fig 1), as basic K49 PLA_2_s constitute nearly half of the entire PLA_2_ content, while *C*. *simus* is the only one of the three snakes containing neurotoxic crotoxins–and only in low abundance (3.8% dry weight) in venom of the adult snakes used in immunization [45]. Moreover, the antivenom has previously been found not to neutralize neurotoxic effects of crotoxins in three investigated *Crotalus* species [34], and not to neutralize a crotoxin-like PLA_2_ heterodimer described in the venom of *Bothriechis nigroviridis* [21].

The signal profiles of the aligned PLA_2_s in Fig 6A reveal recognition of 15-mers in three separated segments. Most (34 out of 60) toxins recognized in the first segment were found to belong to the basic D49 group of which 60% (34 out of 57) were recognized at the N-terminal site. Furthermore, an additional 20 PLA_2_s from the acidic D49 and basic D49 (crotoxin) groups were found to closely resemble the recognized D49 sequences at this site. None of the 6 basic K49 toxins recognized in the segment were found among the top 50 toxins with highest binding signal, showing that most antibodies targeting this site are antibodies targeting D49 PLA_2_s. Three D49 toxins originating from venoms (*B*. *asper* and *L*. *stenophrys*) used in the immunization mixture were included in the study. However, none of the recognized toxins in the first segment originate from *Bothrops* or *Lachesis* snakes, despite that 47 *Bothrops* and 7 *Lachesis* PLA_2_ sequences covered the region. On the other hand, 7 *Crotalus* toxins were among the recognized toxins, indicating that the antibodies targeting this segment are likely to be a result of the presence of *C*. *simus* D49 PLA_2_s in the immunization mixture. A previous study investigated the different recognition profiles of two antivenoms prepared by immunization with either four *Bothrops* species or a *Crotalus* snake [23]. The study involved peptides derived from three individual PLA_2_s (one from each of the non-crotoxin groups) from *B*. *jararacussu* venom using a low-resolution variant of the analysis performed in this study. Although the investigated toxins were of *Bothrops* origin, a high level of binding for the anti-crotalus antivenom was observed at the N-terminal end of the basic D49 PLA_2_. Taken together with our results, this indicates that the N-terminal basic D49 PLA_2_s from *Crotalus* snakes might generally be more immunogenic than the *Bothrops* counterparts.

The second segment in Fig 6 was mainly recognized for a subset of toxins from the acidic D49 group with no toxins originating from the immunization venoms. The origin of the antibody response towards this site is unclear, as both one *Bothrops* enzyme and one *Crotalus* enzyme are recognized here. Due to the low level of information on this linear epitope element, it is not discussed further here.

The third and last segment in Fig 6A, corresponding to the C-terminal residues, was more difficult to define compared to any other linear epitope element identified in the study. As many very different sequences were recognized by the antivenom, the information content of the sequence logo in Fig 6C was low in most positions. Also, the borders of the segment were difficult to define, as the signal profile of the 31 toxins found to be recognized in the area peaked at various positions between peptide number 97 and 196. In extreme cases, significant signals were observed for 13 overlapping peptides, meaning that only 2 residues were shared between all 13 peptides, and that two or more linear epitope elements are likely to exist in the area. In contrast to segment 1, dominated by basic D49 PLA_2_s, 18 of the 31 toxins recognized in segment 3 were found to be members of the basic K49 group. Furthermore, 9 of the recognized toxins belong to the acidic D49 family. Comparing the core sequences of the recognized acidic D49 and the basic K49 toxins, only one lysine was found to be conserved. We therefore conclude that at least two populations of antibodies with very different binding preferences, but recognizing topologically equivalent sites, are likely to exist in the antivenom.

Looking at which species the recognized toxins in segment 3 originate from, both antibodies recognizing K49 and antibodies recognizing acidic D49 PLA_2_s appear to be induced by the *B*. *asper* proteins in the immunization mixture. This conclusion is based on the recognition of 11 *Bothrops* toxins (4 acidic D49 and 7 basic K49 PLA_2_s), including two *B*. *asper* K49 PLA_2_s, while no *Crotalus* or *Lachesis* toxins were recognized in this segment. The anti-*Bothrops* antivenom of the low-resolution study, previously discussed, was also found to recognize peptides in the C-terminal end of K49 PLA_2_s, while the anti-*Crotalus* antivenom did not recognize this part of any of the *Bothrops jararacussu* toxins [23].

The C-terminal region of the basic K49 myotoxins is amphiphilic, contains several positively charged residues, and is known to be critical for toxicity by non-enzymatic disruption of the plasma membrane of skeletal muscle fibers [61]. Antivenom recognition to this site is therefore an example of neutralization by binding directly at the toxic site. Furthermore, the C-terminal region of the *B*. *asper* K49 myotoxin P24605 has also previously been found to be a neutralizing epitope in mice and rabbits [24,25]. As a passing remark, three other batches of the horse antivenom showed poor recognition of the C-terminal region of P24605 [24], which is in agreement with the findings in the present study, where the toxin was the only *B*. *asper* K49 PLA_2_ with a signal profile not passing the significance threshold.

Linear epitope elements were detected in 2 out of the 6 described PLA_2_ sequences from the venoms used for immunization–an intriguing result. It is possible that the 3 *B*. *asper* toxins with signal profiles below the significance threshold are not present in sufficient concentration in the immunization mixture to elicit a strong enough antibody response for this analysis to detect binding. Alternatively, these toxins might contain epitopes with no linear parts (traditionally referred to as “conformational epitopes”). Previously, two neutralizing monoclonal antibodies developed to bind two *B*. *asper* K49 myotoxins were found not to bind the toxins when the toxins had been denatured, thus suggesting discontinuous epitopes [62]. Alternatively, an explanation for the lack of recognized linear element for 4 of the of *B*. *asper* PLA_2_s could be that many PLA_2_s are simply poor immunogens as observed for other PLA_2_s [63].

### Serine proteases are recognized at a site known to induce allosteric inhibition

**Fig 7:**
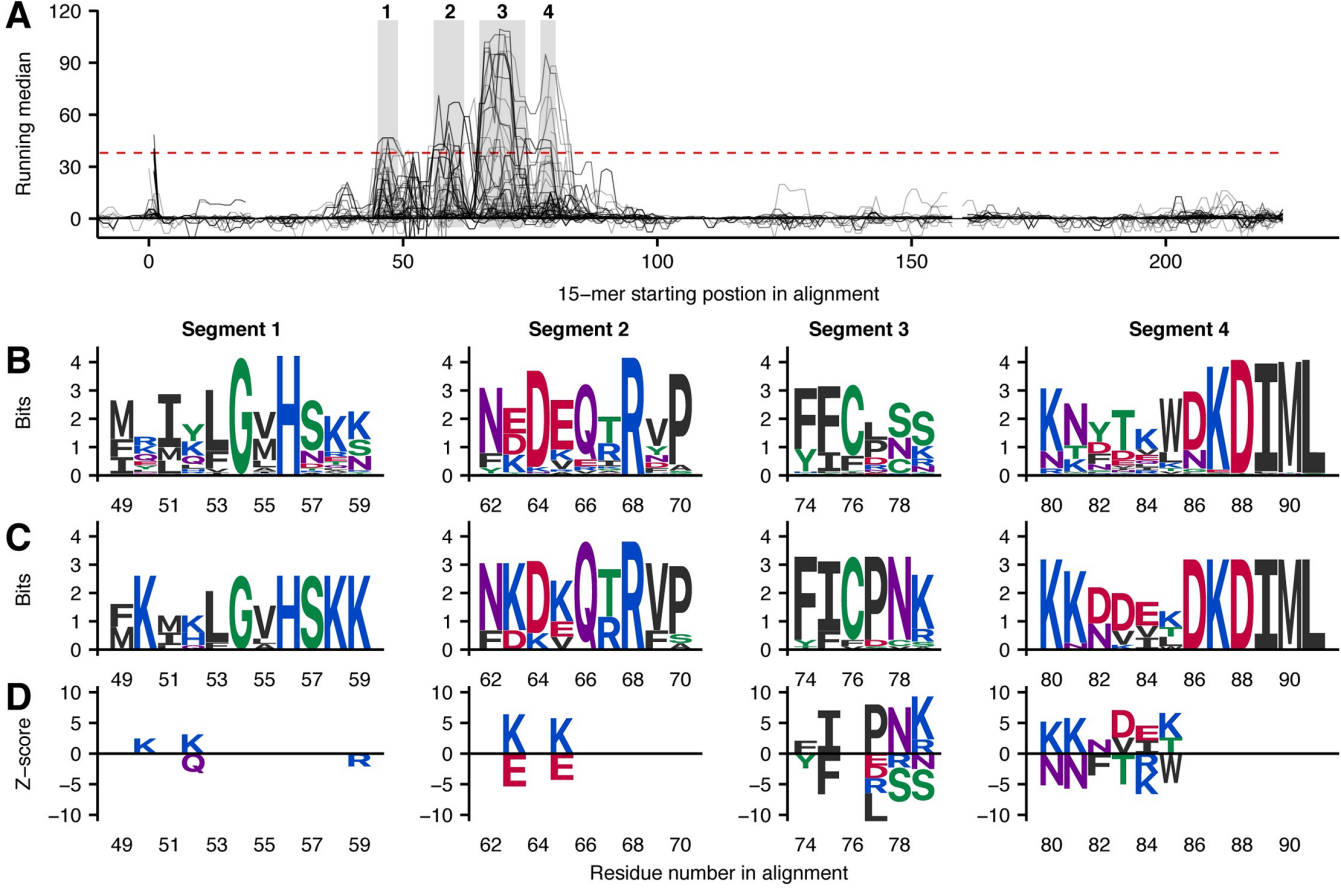
Representations of linear epitope elements in SVSPs. See Fig 3 legend for detailed explanation. (A) Four separate segments subject to antivenom recognition are identified in the primary sequences of 135 SVSPs (20 sequences labelled as partial sequences). All antivenom recognized peptides are found in four segments in close proximity to each other. (B) Sequence logos (Shannon type) representing the overlap between peptides from all toxins in each segment of the multiple sequence alignment. (C) Sequence logos (Shannon type) solely representing toxins recognized in a segment. (D) Z-score sequence logos showing SigniSite [37] output.

SVSPs are enzymes interfering with the blood-clotting system and generating vasoactive mediators from endogenous precursors [64,65]. For many pit vipers, SVSPs are major contributors to venom toxicity, and it is therefore relevant to understand how antivenom antibodies can recognize and neutralize this toxin family. In this study, 135 SVSP sequences were investigated, of which 115 are complete sequences covering the entire enzyme in mature form. Binding of antivenom antibodies to SVSP-derived 15-mer peptides were obtained for more than half (62) of the full-sequence toxins (Fig 1). Mapping of signals from antibody recognition of 15-mer peptides to a multiple sequence alignment of SVSPs shows binding to four individual segments in close proximity to each other (Fig 7).

The core residues of segment 3 and 4 in Fig 7A turned out to border each other (Fig 7B–7D). Further investigation of the results revealed a large overlap between the toxins recognized in these two segments, as 11 out of the 12 SVSPs recognized in segment 4 were also recognized in segment 3, while an additional 18 toxins were solely recognized in segment 3. Of these 11 toxins recognized in both segments, 9 were found to originate from venoms used in the immunization mixture or from related species of the same genera. This finding indicates that antibodies might target two separate linear elements of the toxins used for immunization. As the residues from position 77–84 in the multiple sequence alignment are poorly conserved, and since the recognized residues are generally not among the most frequently observed residues (Fig 7B and 7C), antibody binding to the linear epitope element in segment 4 is easily lost to naturally occurring sequence variation.

**Fig 8:**
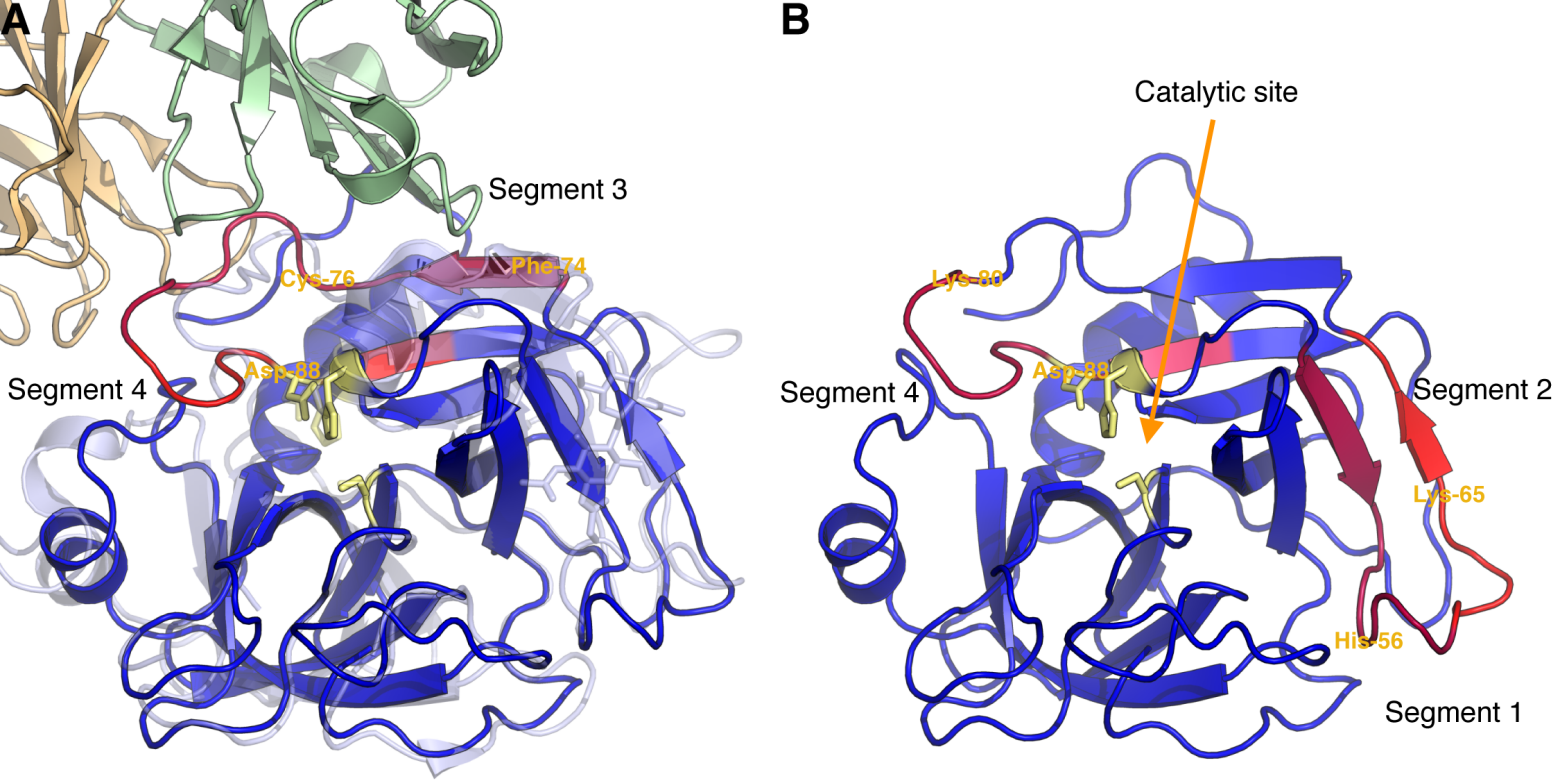
Mapping of linear epitope elements to SVSPs. (A) The SVSP Q072L7 from *L. stenophrys* is recognized in segment 3 and 4. Superposition of a homology model SVSP Q072L7 and the human serine protease HGFA in complex with an allosteric antibody [66]. (B) The SVSP J3S832 from *C. adamanteus* is recognized in segment 1 and 2.

Mapping of the identified linear epitope elements to structural models of SVSPs (Fig 8) reveals that all segments are accessible to antibodies, but also that only segment 4 is overlapping residues of the enzymatic cleft. Binding of antivenom antibodies at the enzymatic site will inhibit function of the aspartic acid of the catalytic triad, thereby neutralizing the toxins. However, binding to segment 3 was more prominent (Fig 7A), and a higher number of toxins were recognized at this site compared to segment 4. A study of antibody binding to a similar site in a human serine protease, the hepatocyte growth factor activator (HGFA), sheds light on a possible mechanism of neutralization [66]. In the study, the said site was found to be an allosteric site, where binding of monoclonal IgG antibodies induced a conformational change incompatible with substrate binding at the enzymatic site. As the overall structures of HGFA and SVSPs are conserved, the reported allosteric mechanism of serine protease inhibition may likely explain how antivenom antibodies neutralize SVSPs by binding to segment 3.

The possible therapeutic relevance of segment 1 and 2 is not possible to assess based on prior studies and it is outside of the scope of this study. Disregarding the neutralization potential of antibodies binding at these two segments, segment 2 was by far the most commonly recognized site of the two, with 27 recognized toxins, compared to 8 toxins in segment 1. A remarkably low number of only 3 toxins were recognized in both segment 2 and segment 3 or 4. This indicates that segment 2 might constitute an alternative epitope, which is mostly found in toxins not recognized in segment 3 or 4. Of the 27 recognized toxins in segment 2, 9 belong to *Crotalus* species, while only one originates from a *Bothrops* species and none from *Lachesis* species. It is therefore likely, that this linear epitope element is a result of immunization with SVSPs from *C*. *simus* venom (present in venom (Fig 1), although no SVSP sequences available from this species).

The high-density peptide microarray methodology employed here does not take post-translational modifications into account. For most members of the PLA_2_ family this is not an issue as they rarely have post-translational modifications [67]. However, PII and PIII SVMPs, and especially SVSPs are commonly N-glycosylated. In extreme cases in SVSPs, sugar moieties can constitute more than half of the molecular mass [64,68]. Yet, none of the recognized toxins contain an asparagine residue subject to glycosylation in any of the antibody binding peptides identified here, meaning that the data should contain no false positives from this effect. N-glycosylation is frequently found across SVSPs in alignment position number 20, 81, 99, 100, 132, 148, and 231. This could potentially explain the complete lack of antibody recognition to the second half of the alignment, as the horse antibodies may recognize epitopes comprising the foreign (snake-type) N-glycosylations and not the “naked” peptides.

Focusing on the well-recognized segments 3 and 4, for which mechanisms of neutralization can be deduced, their high variability may have large implications for obtaining a broad-acting pit viper antivenom. Both traditional antivenom production, developments within novel immunization strategies employing venoms, recombinant toxins, synthetic peptides, or even DNA/RNA immunization techniques, and development of novel recombinant snakebite therapies based on mixtures of monoclonal antibodies [7] may need to bind a diverse set of toxins with a high degree of amino acid variation in these sequence segments. Therefore, it is likely that multiple different antibodies are needed to target and neutralize an entire group of toxins in these strategic sites. However, future studies with all the identified linear epitope elements are needed to verify if they correspond to neutralizing epitopes.

### Conclusion

The data presented here reveals important molecular details for understanding the paratope-epitope interactions between antivenoms and linear elements of viperid venom toxins, and contributes to explain the cross-reactivity, i.e. paraspecific recognition, often observed between antibodies and venom proteins from species not included in the immunization mixture.

Most of the venom protein families discussed in this work are enzymes, and they either exert their toxic effects locally, in the case of SVMPs and PLA_2_s that induce local hemorrhage and necrosis, or systemically, as in the case of enzymes that act on clotting factors or SVMPs that induce systemic hemorrhage. These toxins are readily recognized by antivenom antibodies at various segments of their sequence, but seldom in their “catalytic site”, which in these enzymes is largely responsible for toxicity. This reveals an important difference between the SVMP and PLA_2_ toxin families and neurotoxins from the three-finger toxin (3FTx) and Kunitz-type inhibitor (including dendrotoxins) families. Whereas the latter families are recognized by antivenoms in their toxic sites [32], most of the linear epitope elements of the dominant viperid toxin enzyme families are found in sites different from the catalytic site. This could suggest that these enzymatic toxins may be neutralized via other effects such as steric hindrance or allosteric effects. Alternatively, since enzymatic toxins often have molecular regions, which could be exosites, that enable them to recognize relevant tissue targets [53,57,58], it is likely that antibodies recognizing epitopes outside the catalytic site may be *de facto* neutralizing. Binding to exosites may preclude interaction between these toxic enzymes and their targets in the plasma membrane of cells, extracellular matrix proteins, or blood clotting factors, even if their catalytic sites are not blocked or disrupted. Likewise, in the case of toxic PLA_2_ homologues devoid of enzymatic activity, antibody neutralization occurs through the binding of regions, which determine the ability of toxins to interact with targets in the muscle cell plasma membrane, as in the case of K49 PLA_2_s. It is also possibly that, instead of inhibiting enzymatic activity, neutralizing antibodies derive their therapeutic effects via the removal of toxin-antibody complexes through endocytosis by immune cells [69]. However, the extent of such neutralization mechanism is not possible to address based on the current study.

Our observations highlight a complex molecular immunological scenario for snake venom toxins, in which antivenom antibodies may exert their neutralizing ability by several mechanisms depending on the nature of the toxin family.
